# Dermoscopy for monitoring therapeutic response to hybrid cooperative complexes of hyaluronic acid in women with vulvar lichen sclerosus and atrophy

**DOI:** 10.3389/fmed.2025.1540428

**Published:** 2025-10-13

**Authors:** Giulio Rizzetto, Edoardo De Simoni, Alessandro Borghi, Elisa Molinelli, Marinella Tedesco, Emilia Migliano, Corrado Tagliati, Monica Corazza, Franco Grimolizzi, Gilberto Bellia, Camilla Barduagni, Annamaria Offidani, Oriana Simonetti

**Affiliations:** ^1^Dermatological Clinic, Department of Clinical and Molecular Sciences, Polytechnic Marche University, Ancona, Italy; ^2^Department of Medical Sciences, Section of Dermatology and Infectious Diseases, University of Ferrara, Ferrara, Italy; ^3^Department of Plastic and Regenerative Surgery, San Gallicano Dermatological Institute IRCCS, Rome, Italy; ^4^IBSA Farmaceutici Italia Srl, Lodi, Italy

**Keywords:** vulvar lichen sclerosus, hyaluronic acid, hybrid cooperative complexes, videodermatoscopy, ultrasound

## Abstract

**Introduction:**

Vulvar Lichen Sclerosus (VLS) is a chronic relapsing inflammatory disease involving the anogenital region, resulting in vulvar atrophy and distressing symptoms. A preliminary prospective observational study was conducted to investigate the efficacy of hybrid cooperative complexes (HCC) of low- and high-molecular weight hyaluronic acid (HA) for the treatment of female patients with vulvar atrophy and lichen sclerosus.

**Methods:**

Female patients with coexisting vulvar atrophy and lichen sclerosus (*N*=15) received two HCC injections at 32 mg/ml (one month apart). At baseline, 1-month, 3-months, and 6-months post-treatment, patients were assessed for reference dermatoscopic parameters using videodermatoscopy, symptoms, quality of life (Dermatology Life Quality Index [DLQI]) and sexual function (Female Sexual Function Index [FSFI]). The hypoechoic band was evaluated using ultrasound.

**Results:**

Both DLQI and FSFI scores improved with HCC treatment, with significant improvements at 1-month, 3-months and 6-months post-treatment vs baseline (*p*<0.05 for all). The thickness, homogeneity and smoothness of the hypoechoic band increased post-treatment. No side effects or complications were reported. The reference dermatoscopic features of vascularisation, blue grey dots, purpuric lesions, horny pearls, scales, ice silvers structures or whitish background were reduced at 6-months post-treatment vs baseline; significant reductions were observed for scales (baseline vs 1-month and 6-months post-treatment; *p*<0.05 for both). Scores for pain, itching, and burning were reduced at 1-month, 3-months and 6-months post-treatment vs baseline (*p*<0.05 for all).

**Discussion:**

These preliminary results add to the growing body of evidence highlighting the promising efficacy of HCC of HA for the treatment of VLS.

## 1 Introduction

Vulvar Lichen Sclerosus (VLS) is a chronic relapsing inflammatory disease which commonly affects the anogenital areas of females (1, 2). Diagnosis is usually based on clinical examination, with typical clinical signs including whitening and atrophy of the skin, which may present as a figure-of-8 pattern involving the vulva, perineum, and perianal area (1–3). Inflammation leads to scarring and anatomical changes such as clitoral phimosis, labial minor adhesions with different degrees of atrophy and vulvar introital stenosis (1, 2). Common symptoms include intense vulvar pruritus, burning pain, painful or less pleasurable sexual intercourse and anal or genital bleeding due to fissuring of the damaged tissue (1–3). Despite the distressing symptoms the clinical diagnosis of VLS may be delayed by up to 5 years, potentially due to reasons such as delay in seeking advice, unfamiliarity with the condition and misdiagnosis (4). Additionally, women with VLS have an estimated 2–5% lifetime risk of developing squamous cell carcinoma, and up to 65% of vulvar cancers occur in individuals with a history of VLS (5).

The cause of lichen sclerosus is unknown, though research has found links to genetic, environmental, hormonal, and immune-related factors (1). Although it can arise at any age, it commonly occurs in the prepuberty, perimenopause or postmenopause periods (1). The exact prevalence of VLS is unknown, but suspected prevalence varies between 0.1% in children and 3% post-menopausal women (5). The incidence of VLS may be rising; analysis of a Dutch pathology registry indicated that the incidence rate increased from 7.4 per 100,000 woman-years in 1991 to 14.6 per 100,000 woman-years in 2011 (6).

Potent to very potent topical corticosteroids, such as topical clobetasol propionate, are the treatment of choice for VLS (1). However, some patients may be intolerant, fail to respond to treatment and a small proportion may experience side effects such as dermal atrophy and dermatitis after prolonged use (>12 weeks) (7, 8). When disease control is achieved, corticosteroids or moisturisers are used as maintenance treatment (4). However, not all patients can achieve disease control and there are several options for treatment-resistant disease; these include topical and oral retinoids, steroid injections, calcineurin inhibitors (such as tacrolimus and pimecrolimus), methotrexate and hydroxyurea, with some of these treatments associated with severe side-effects. Surgical intervention may be required for patients with malignancy or post-inflammatory sequelae (1, 4). Autologous platelet-rich plasma and autologous fat injection (lipofilling) have been investigated in preliminary studies as a treatment for VLS (9, 10). However, these therapies are unsuitable for some patients, including those with platelet disorder, coagulation disorders and those taking anticoagulant or fibrinolytic drug therapy (11, 12).

Hyaluronic acid (HA)-based dermal fillers have been investigated for the treatment of VLS in two prospective preliminary studies which utilised a novel formulation of HA composed by hybrid cooperative complexes (HCC) of low- and high-molecular weight HA based on patented NAHYCO™ technology. In the first study, 22 patients with VLS were injected with HCC every month for 6 months and resulted in significant improvements in disease symptoms and quality of life (QoL) versus baseline (13). In the other study, 30 patients with VLS received two HCC injections 1 month apart, and experienced a trend toward reduced pain, itching and a significant reduction in dryness at 6 months versus baseline, while sexual function was also improved (14). No side effects or complications were reported in either study. Studies have shown that HCC of HA can stimulate elastin and collagen expression by keratinocytes and fibroblasts *in vitro*, contributing to ameliorating skin extracellular matrix and global homeostasis (14, 15).

To further investigate the benefit of HA for VLS, a preliminary prospective observational study was conducted to investigate the efficacy of HCC of low- and high-molecular weight HA for the treatment of female patients with vulvar atrophy and lichen sclerosus.

## 2 Methods and materials

### 2.1 Study design

**Figure 1:**
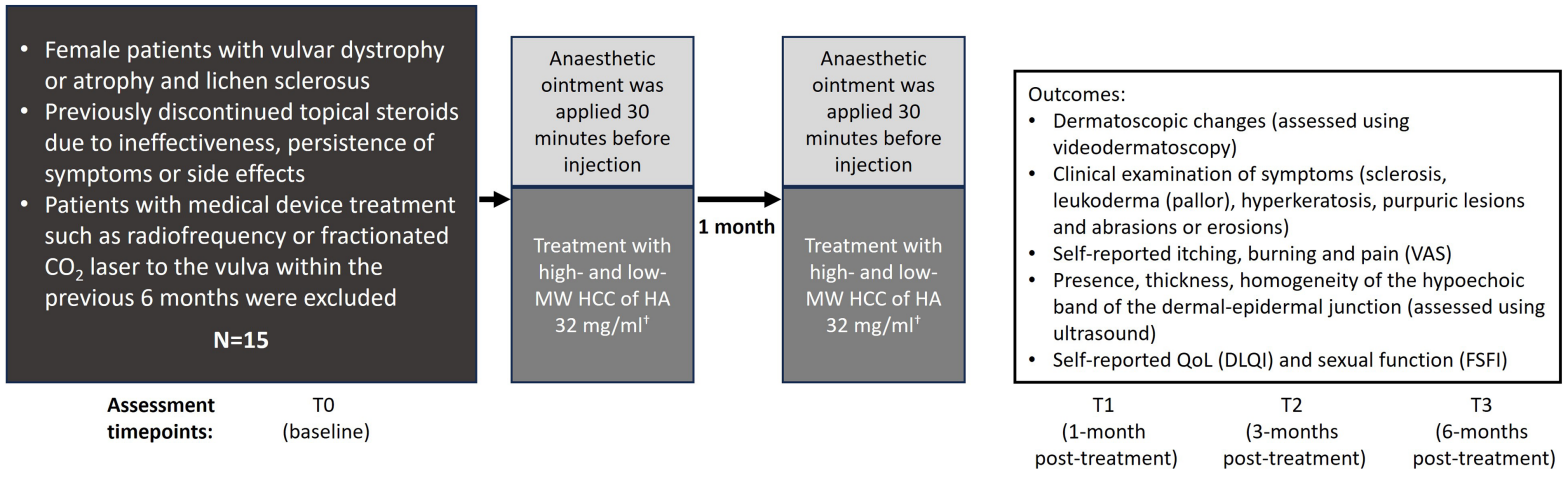
Study design. A single syringe containing 2 mL of HCC was used per treatment and injected into the genital region with a 29-gauge needle and an intradermal wheal technique (0.25 mL per injection site) distributed as follows: two into the posterior fourchette, two into the labia minora, two into the vulvar vestibule, and two near the clitoris. DLQI, Dermatology Life Quality Index; FSFI, Female Sexual Function Index; HA, hyaluronic acid; HCC, Hybrid Cooperative Complexes; MW, molecular weight; N, number of patients; T, timepoint; VAS, Visual Analogue Scale.

This prospective observational study was conducted at the Ancona Dermatology Clinic (Dermatological Clinic, Department of Clinical and Molecular Sciences, Polytechnic Marche University) in collaboration with the Ferrara Dermatology Clinic (Department of Medical Sciences, Section of Dermatology and Infectious Diseases, University of Ferrara) between May 2023 and January 2024, enrolling 15 female patients with vulvar atrophy and lichen sclerosus. This is an ongoing study with further patient enrolment expected. Patients received a highly purified, thermally stabilised, high- and low-molecular-weight HCC of HA of non-animal origin without any chemical crosslinking agents at a dose of 32 mg/mL (IBSA Farmaceutici Italia Srl). Patients received HCC intradermal injections at two separate visits, 1 month apart (Figure 1). Eligible patients were female, aged between 18 and 85 years with signs and symptoms of vulvar lichen sclerosus and atrophy (vulvar dryness, loss of volume and elasticity of labia majora and labia minora, dyspareunia) and who discontinued topical steroid therapy due to ineffectiveness, persistence of symptoms (itching, burning, pain), or the onset of side effects such as thinning of the vulvar skin and telangiectasias. Patients were excluded if they were contraindicated for HA therapy (pregnancy, active infection, history of vulvar neoplasia in the last 5 years) or had previous application of other medical devices such as radiofrequency or fractionated CO_2_ laser to the vulva within the last 6 months. Approval for the treatment of VLS using HCC of HA (IBSA Farmaceutici Italia Srl) was previously obtained from a local ethics committee (Protocol number, Prot.2023 96, 18 May 2023). The study was performed in accordance with the Consolidated Standards of Reporting Trials and the Declaration of Helsinki. All patients signed an informed consent after receiving detailed explanations of the procedure and possible side effects and complications.

### 2.2 Procedures

An anaesthetic ointment was applied to the genital region 30 min before injection. A single syringe containing 2 mL of HCC was used per treatment and injected into the genital region with a 29-gauge needle and an intradermal wheal technique (0.25 mL per injection site) distributed as follows: two into the posterior fourchette, two into the labia minora, two into the vulvar vestibule, and two near the clitoris.

### 2.3 Outcomes, data collection and follow up

The primary objective was improvement of reference dermatoscopic parameters 6 months after treatment with HCC of HA. Secondary objectives included improvement of symptoms and QoL 6 months after treatment with HCC. At baseline, enrolled patients underwent physical examination, including assessment with Dermatology Life Quality Index (DLQI), Female Sexual Function Index (FSFI), clinical, ultrasound and videodermatoscopy assessment before receiving therapy and were re-evaluated at 1-month, 3-months and 6-months post-therapy. Patients were followed for an additional 6 months after the final evaluation.

Dermatoscopic changes were assessed using videodermatoscopy (FotoFinder® medicam 1,000). Photos were taken in the same anatomical locations during follow-up, using minimal pressure to preserve the morphology of the vessels. Parameters evaluated included vascularisation, whitish plaques without structure, blue-grey dots/globules with a peppering pattern, comedone-like outlets, scales, structures similar to “ice silvers”/"bright white streaks,” whitish background, purpuric globules and erosions. Photos were taken at 20x and 40x magnification, and always taken by the same dermatologist for consistency. For parameter assessment, the dermatoscopic photos were examined in a blinded manner by two different dermatologists; a third dermatologist was consulted in the event of conflicting opinion and resolved by collective decision. Symptoms were assessed by clinical examination and included sclerosis, leukoderma (pallor), hyperkeratosis, purpuric lesions and abrasions or erosions, itching, burning and pain. The evaluation of clinical and dermatoscopic parameters was graded according to a 4-value scale (0, none; 1, mild; 2, moderate; 3, severe); itching, burning and pain were measured using the visual analogue scale (VAS; 0 = no symptom impact, 10 = worst impact imaginable). The morphology, thickness and homogeneity of the hypoechoic band of the dermal-epidermal junction was assessed using ultrasound (Esaote myLab™ ultrasound machine with a 15–20 MHz probe). QoL was evaluated using the dermatology specific DLQI (minimum score = 0, indicates no impact on QoL; maximum score = 30, severe impact on QoL) and the FSFI which evaluates desire, arousal, lubrication, orgasm, satisfaction, and pain domains (score range: 2–36, with lower scores corresponding to worse sexual functioning).

### 2.4 Statistical analysis

Results were reported as mean and standard deviation for numerical variables, while percentages were used for categorical variables. Quantitative pre- and post-treatment values assigned to each clinical and dermatoscopic parameter were compared using a paired t-test when data were normally distributed, otherwise Wilcoxon signed-rank test was used. A binomial test with one-tailed t-test was used to compare dichotomous variables. *p* < 0.05 was considered statistically significant for all tests.

## 3 Results

### 3.1 Baseline demographic and clinical characteristics

A total of 15 female patients with vulvar atrophy and lichen sclerosus were enrolled and received treatment; all 15 patients were available for assessment at each post-treatment assessment. Mean patient age was 57 and most patients had disease symptoms at baseline, which included sclerosis, leukoderma (pallor), hyperkeratosis, purpuric lesions and abrasions or erosions, itching, burning and pain. A detailed description of baseline demographic and clinical characteristics is presented in Table 1. No complications or side effects were reported in the study.

**Table 1:** Baseline demographic and clinical characteristics.

Characteristic	Female patients, *N* = 15
Mean age (range)	57 (47–69)
Signs and symptoms, *n* (%)	
Sclerosis	15 (100)
Leukoderma (pallor)	15 (100)
Hyperkeratosis	7 (47)
Purpuric lesions, abrasions or erosions	13 (87)
Itching	14 (93)
Burning	10 (67)
Pain	12 (80)
Mean DLQI score (SD)	8 (5.5)
Mean FSFI score (SD)	13 (10.1)
Previous treatments	
Topical clobetasol propionate	15 (100)
Topical calcineurin inhibitor	3 (20)

DLQI, Dermatology Life Quality Index; FSFI, Female Sexual Function Index; SD, standard deviation.

### 3.2 Dermatoscopic features

**Figure 2:**
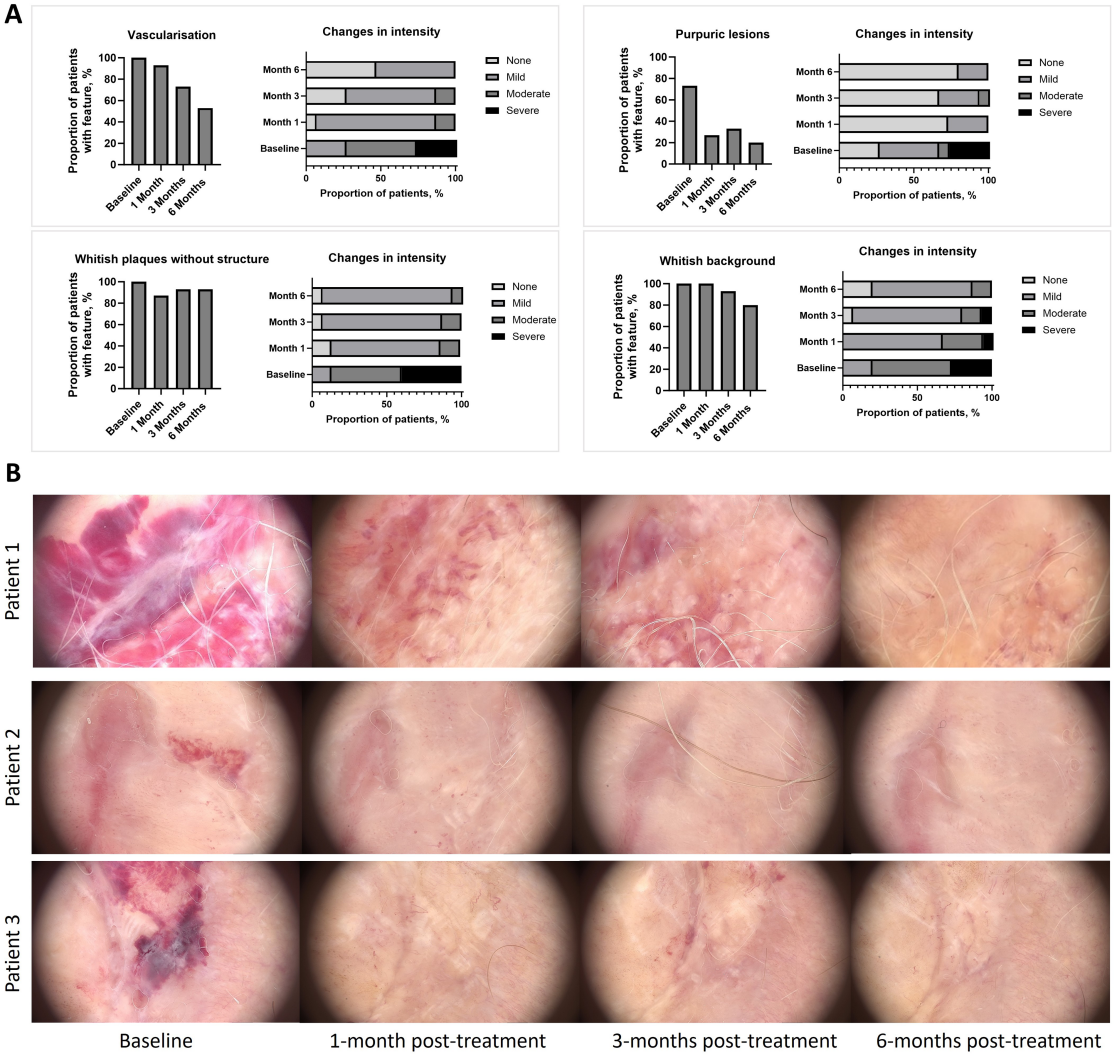
**(A)** Improvement of reference dermatoscopic features versus baseline, **(B)** Dermatoscopic images taken by videodermatoscopy. Dermoscopy images showing the therapeutic response in three representative patients with active lichen sclerosus at baseline, 1 month, 3 months, and 6 months post-treatment. In Patient 1, baseline shows diffuse purpuric lesions and a whitish background. At 1 month post-treatment, there is complete resolution of purpuric lesions and a significant reduction in the whitish background. By 3 months, residual lesions are minimal, and at 6 months, the skin appears fully restored. In Patient 2, baseline presents reddish-purple discoloration with inflammation. At 1 month, redness and inflammation have significantly decreased, with further improvement by 3 months. At 6 months, the skin looks almost normal, with near-complete resolution. In Patient 3, baseline shows dark purple discoloration and marked inflammation. After 1 month, there is visible improvement in pigmentation and inflammation, with further resolution by 3 months. By 6 months, the skin is nearly fully restored. This figure illustrates the progressive improvement and resolution of lichen sclerosus symptoms in these representative cases following treatment.

All the reference dermatoscopic features showed a trend towards reduction at 6-months post-treatment when compared with baseline (Figure 2 and Supplementary Table 1). Significant reductions were observed for scales [baseline vs. 1-month post-treatment and vs. 6-months post-treatment; *p* < 0.05 for both comparisons (Supplementary Table 2)]. Across the assessment points, patients experienced a reduction in the intensity of dermatoscopic feature versus baseline typically moving from severe or moderate intensity to mild intensity or absence of the feature. Reduction in intensity was observed for vascularisation, whitish plaques without structure, blue grey dots, purpuric lesions, horny pearls, scales, ice silvers structure, comedone-like outlets and whitish background (Figure 2A and Supplementary Table 1). Figure 2B illustrates visual improvements in diffuse purpuric lesions at 1-month, 3-months and 6-months post-treatment compared with baseline.

### 3.3 Assessment of signs, clinical features and symptoms

**Figure 3:**
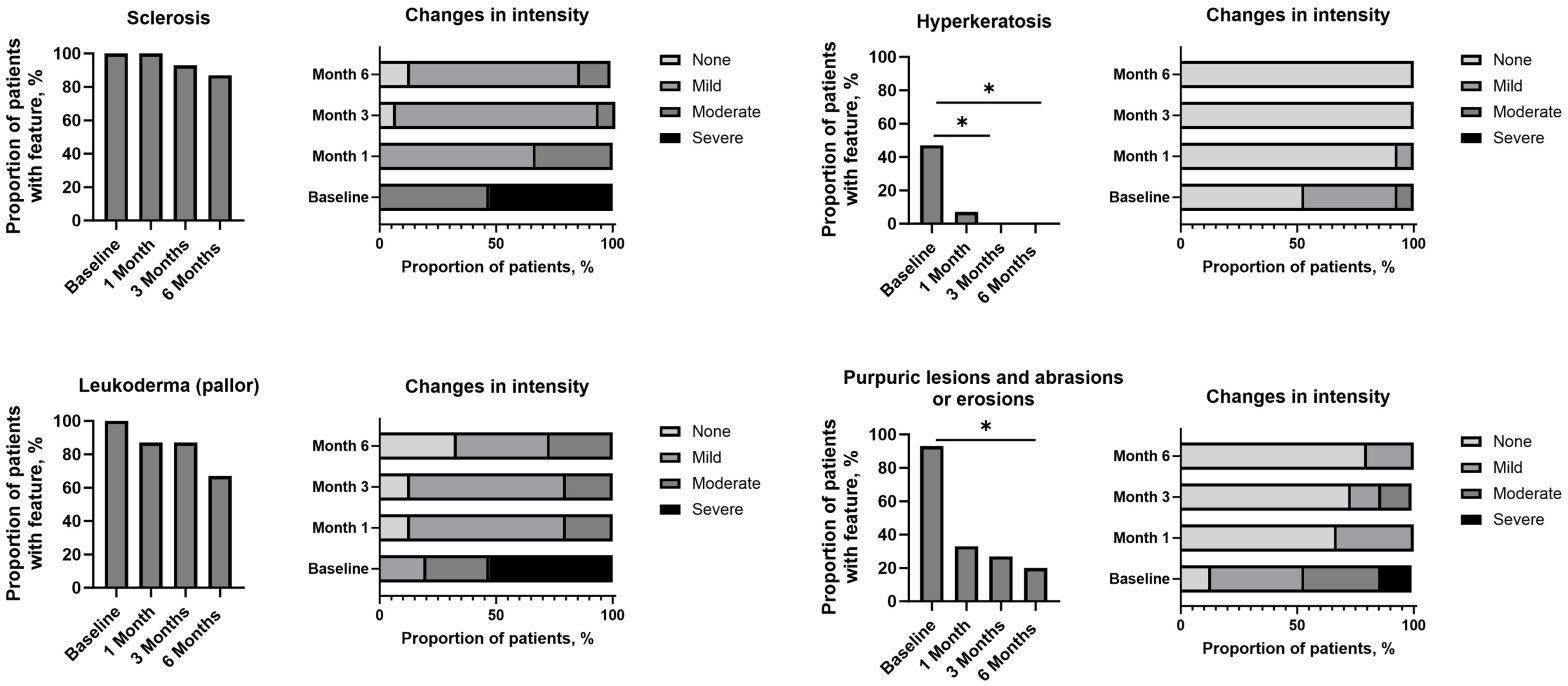
Improvement in signs/clinical features based on clinical examination versus baseline. Analysis conducted using a binomial test with one-tailed *t*-test: * *p* < 0.05.

**Figure 4:**
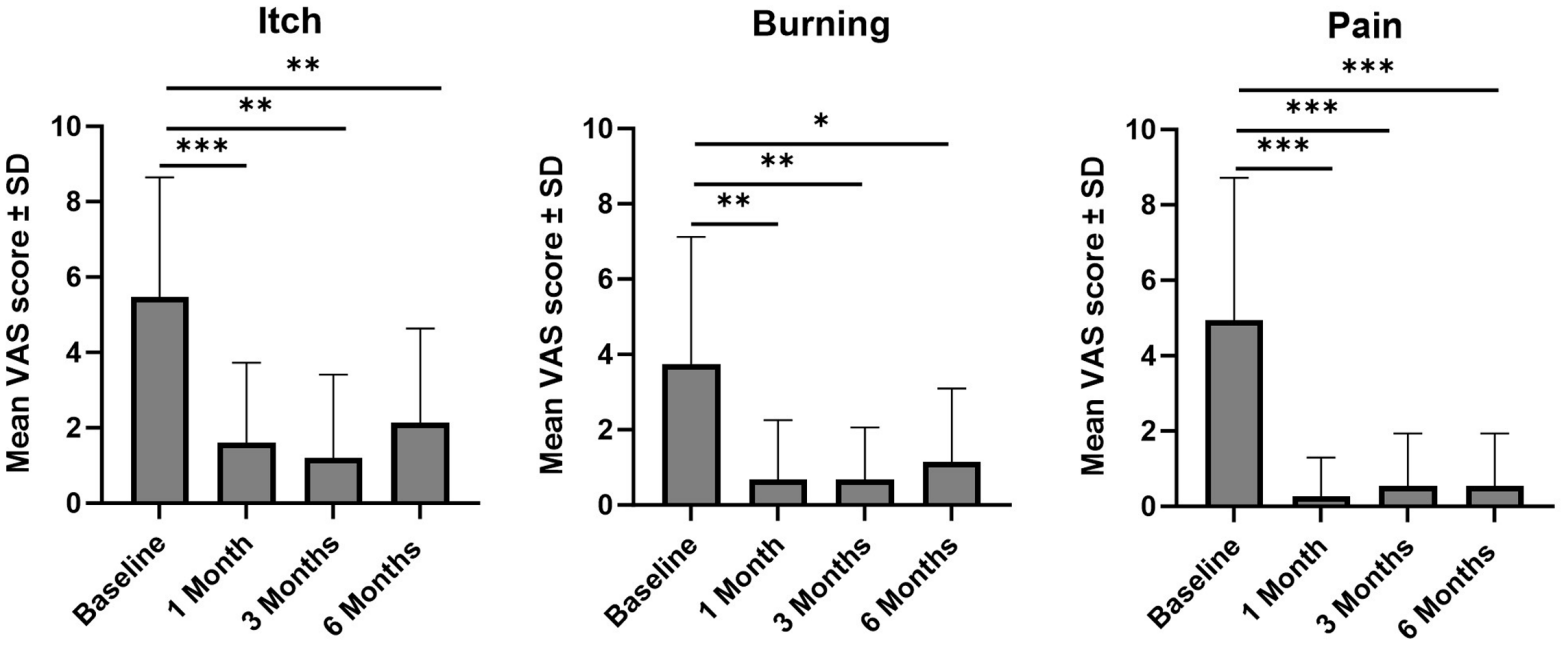
Improvement in itch, burning and pain (mean VAS score ± SD) versus baseline. Analysis conducted using Wilcoxon matched-pairs signed rank test: *p < 0.05, **p < 0.01, ***p < 0.001. SD, standard deviation; VAS, visual analogue scale.

All clinically assessed signs and clinical features, namely sclerosis, leukoderma, hyperkeratosis, or purpuric lesions and abrasions or erosions, showed a trend towards reduction at 6-months post-treatment when compared with baseline (Figure 3). A significant reduction in proportion of patients with hyperkeratosis was observed at 3-months and 6-months post-treatment vs. baseline (*p* < 0.05 for both), with a significant reduction also observed for purpuric lesions and abrasions or erosions observed at 6-months post-treatment versus baseline (*p* < 0.05). Across the assessment points, patients experienced a reduction in the intensity of sclerosis, leukoderma, hyperkeratosis, or purpuric lesions and abrasions or erosions versus baseline, typically moving from severe or moderate intensity to mild intensity or absence of the feature (Figure 3). Additional comparisons of sclerosis, leukoderma, hyperkeratosis, or purpuric lesions and abrasions or erosions at different time points are available in Supplementary Table 3. Mean VAS scores were significantly reduced at 1-month, 3-months and 6-months post-treatment versus baseline for the symptoms of itch (1.6, 1.2, 2.1 vs. 5.5, respectively), pain (0.3, 0.5, 0.5 vs. 4.9), and burning (0.7, 0.7, 1.1 vs. 3.7) (all *p* < 0.05; Figure 4).

### 3.4 Ultrasound evaluation of the hypoechoic band

**Figure 5:**
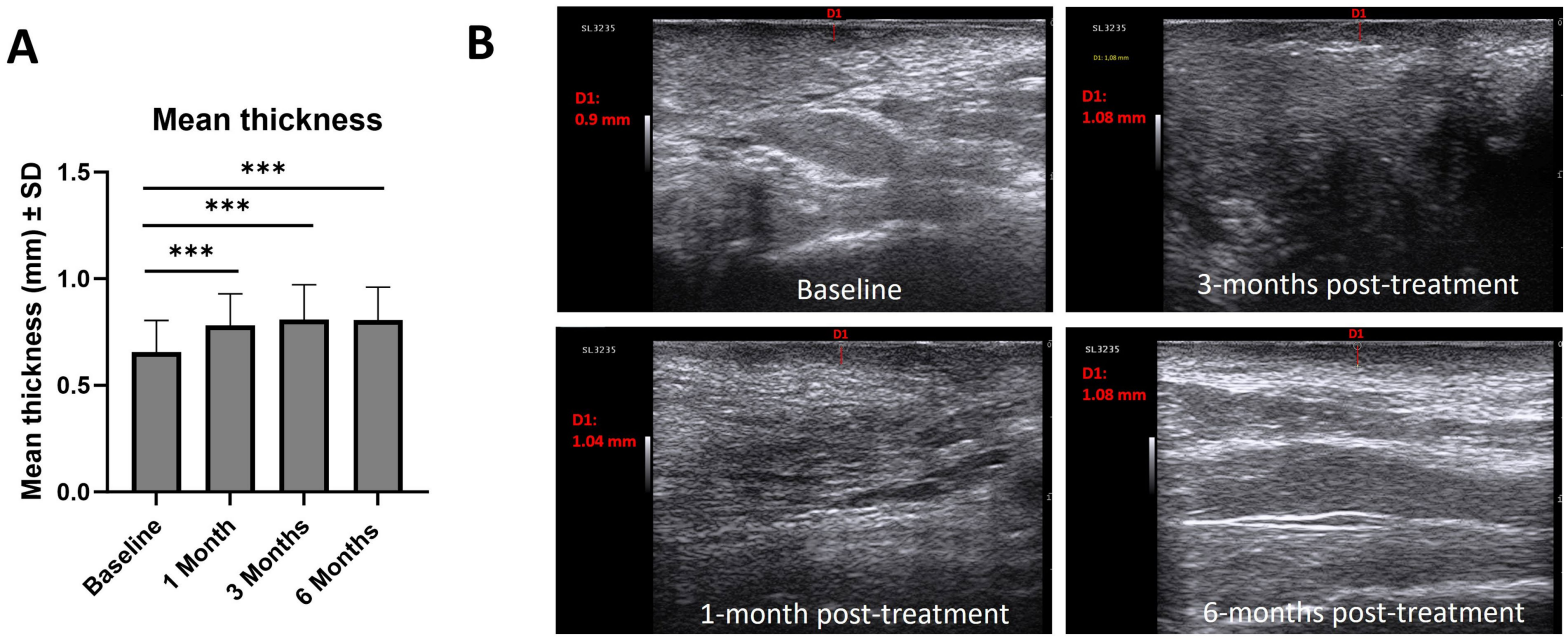
**(A)** Mean thickness (mm, ± SD) of the hypoechoic band of the dermal-epidermal junction and ultrasound images of the hypoechoic band, **(B)**. Example of 3 ultrasound measurements of hypoechoic band thickness. Analysis conducted using Wilcoxon matched-pairs signed rank test: *p < 0.05, **p < 0.01, ***p < 0.001. SD, standard deviation.

The thickness of the hypoechoic band of the dermal-epidermal junction increased from baseline (0.66 mm) at 1-month (0.78 mm; *p* < 0.001), 3-months (0.80 mm; p < 0.001) and 6-months post-treatment (0.80; p < 0.001) (Figure 5A). Regarding epidermal morphology of the hypoechoic band, the proportion of patients with smooth morphology increased after treatment, while irregular morphology decreased [baseline (smooth/irregular, 7%/93%), 1-month post-treatment (40%/60%), 3-months post-treatment (67%/33%), 6-months post-treatment (73%/27%)]. Similarly, the homogeneity of the of the hypoechoic band increased after treatment [baseline (homogeneous/non-homogeneous, 0%/100%), 1-month post-treatment (40%/60%), 3-months post-treatment (67%/33%), 6-months post-treatment (47%/53%)]. Figure 5B provides illustrative ultrasound images which indicate an increase in hypoechoic band thickness from baseline compared with 1-month and 3-months post-treatment.

### 3.5 Quality of life and self-reported sexual function

QoL improved after treatment. The mean DLQI score was lower at 1-month, 3-months and 6-months post-treatment vs. baseline (4.5, 4.1, 4 vs. 8.0, respectively; Wilcoxon signed-rank test, *p* < 0.05 for all comparisons). Similarly, sexual functioning improved after treatment, with increased FSFI scores observed at 1-month, 3-months and 6-months post-treatment vs. baseline (16.1, 16.9, 17.6 vs. 13.5; paired *t*-test, *p* < 0.05 for all comparisons). At baseline, more than half of the patients (*n* = 8) were not sexually active. At 6-months post-treatment, all patients were sexually active, likely due to an improvement in symptoms.

## 4 Discussion

This preliminary, prospective study demonstrated that in females with vulvar atrophy, and lichen sclerosus, treatment with high- and low-molecular weight hyaluronic acid hybrid cooperative complexes (HCC of HA) was associated with significant reductions in dermatoscopic features of disease and other clinically assessed symptoms compared to baseline. These features and symptoms typically shifted from severe or moderate intensity to mild or absent. Statistically significant improvements were observed in key symptoms such as itching, burning, and pain, alongside enhanced quality of life (QoL) and sexual function. Importantly, no side effects or complications were reported during the study. Primary objective was improvement of reference dermatoscopic parameters, as assessed by videodermatoscopy. Videodermatoscopy is a non-invasive, routinely used method that can allow integrative monitoring of skin trophism conditions, including patients with lichen sclerosus (16). This method has already been used to assess dermatoscopic changes after topical steroid therapy in patients with VLS and has been shown to be useful in monitoring treatment response (17). The majority of dermatoscopic features assessed in this study have previously been established as features of VLS in previous studies (16, 17). Overall, there was a trend for a reduction in the proportion of patients experiencing dermatoscopic features of disease, particularly vascularisation, blue grey dots, purpuric lesions, horny pearls, ice silvers structures, scales or whitish background at 3-months and 6-months post-treatment versus baseline, indicative that treatment with HCC of HA improved typical dermatoscopic features of disease. Whitish plaques without structure and comedone-like outlets were the only dermatoscopic features where a limited reduction was observed after treatment. However, for whitish plaques without structure and comedone-like outlets, feature severity shifted from severe or moderate intensity to mild intensity or absence of the feature/symptom, indicative of a treatment effect. Of note, in a study assessing dermatoscopic features in VLS after 12-week treatment with topical corticosteroids, comedone-like outlets did not change significantly throughout the treatment (17). It is possible that these dermatoscopic features may be difficult to treat disease features and represents an area for further investigation.

VLS is characterised by common and distressing symptoms such as itching burning and pain, as well as exerting a negative impact on QoL and sexual functioning (1–3). Importantly, study results indicated that treatment with HCC of HA resulted significant improvement in these parameters. Although these are preliminary results with further investigation required, improvement in these important areas highlights the potential value of HCC of HA for the management of VLS. The positive results observed in this study, particularly the significant improvements in symptoms such as itch, pain, burning and QoL and sexual functioning, have also been reported in other studies investigating HCC of HA for the treatment of VLS. In the study by Tedesco et al. (13), 22 patients with VLS were injected with HCC every month for 6 months and resulted in significant improvements in disease symptoms such as itching, pain, burning and QoL versus baseline. In another study, 30 patients with VLS received two HCC injections 1 month apart, and experienced a trend toward reduced pain, itching and a significant reduction in dryness at 6 months versus baseline, while sexual function also improved (14). The results of this preliminary study add to the growing body of evidence highlighting the benefits of HCC of HA for the treatment of VLS.

In our study we evaluated the 6-month response to HCC therapy in patients with lichen sclerosus. However, the follow-up of patients will continue up to 12 months. During the 6 months following the 2 scheduled HCC injections, no further injections were performed. Considering the data relating to previous study about vulvar HCC (13), we expect a duration of the regenerative action of approximately 6 months, however the actual durability of the regenerative effect is not easily quantifiable, also considering different personal response. In some patients the duration of action of HCC could extend up to 12 months, but further studies are needed. We hypothesize that a therapeutic protocol could consist of repeating an injection of HCC after 6 months from the last treatment. However, even the use of higher or more frequent doses could lead to further improvements in terms of tissue regeneration.

Non-invasive assessment is important for VLS, providing dermatologists with morphological information as well as measurable parameters (18). In this study, non-invasive ultrasound evaluation indicated increases in thickness of the hypoechoic band of the dermal-epidermal junction and increasing smooth morphology and homogeneity at 1-month, 3-months and 6-months post-treatment versus baseline. Although beyond the scope of this study, assessing the correlation between hypoechoic band thickness and inflammatory cell infiltration depth in histopathology represents an area for future research for studies of HCC of HA for the management of VLS (18). Furthermore, while significant improvements in some disease features were observed in this study (scales, hyperkeratosis and purpuric lesions, abrasions or erosions at baseline), the sample size was limited to the number of patients with a specific disease feature at baseline. In future studies, enrolling a larger number of patients with a pre-defined disease feature will provide greater statistical power to assess treatment effects.

Despite the encouraging results, several limitations must be acknowledged, including the small sample size, lack of a comparative treatment group, and absence of long-term outcome data. Future studies with larger, more diverse patient populations, and predefined disease features will provide greater statistical power and allow for a more comprehensive evaluation of treatment effects.

The findings from this preliminary study are promising, as the treatment of females with vulvar atrophy, and lichen sclerosus using high- and low-molecular weight hyaluronic acid hybrid cooperative complexes was associated with a reduction in dermatoscopic features of disease, alleviation of common symptoms, and improvements in quality of life and sexual function. Notably, no side effects or complications were observed during the study.

## Data Availability

The raw data supporting the conclusions of this article will be made available by the authors, without undue reservation.
