# Supplementary material for: Dermoscopy for monitoring therapeutic response to hybrid cooperative complexes of hyaluronic acid in women with vulvar lichen sclerosus and atrophy
Source: Front Med (Lausanne). 2025 Oct 13;12:1540428. doi: 10.3389/fmed.2025.1540428 (PMC12558151; doi:10.3389/fmed.2025.1540428)
Supplement: Supplementary file 1 [file Table_1.docx]

Supplementary Material

# Supplementary Table 1: Improvement of additional reference dermatoscopic features versus baseline, including changes in feature intensity.

| Feature | Proportion of patients with feature, % (n) | | | |
| --- | --- | --- | --- | --- |
|  | Baseline (N=15) | 1 month (N=15) | 3 months (N=15) | 6 months (N=15) |
| Blue grey dots | 27 (4) | 13 (2) | 13 (2) | 7 (1) |
| Comedone-like outlets | 40 (6) | 40 (6) | 40 (6) | 27 (4) |
| Horny pearls | 80 (12) | 80 (12) | 53 (8) | 67 (10) |
| Scales | 53 (8) | 7 (1) | 20 (3) | 0 (0) |
| Ice silvers structures | 67 (10) | 47 (7) | 47 (7) | 40 (6) |
| Proportion of patients by feature intensity, % (n) | | | | |
|  | Baseline (N=15) | 1 month (N=15) | 3 months (N=15) | 6 months (N=15) |
| Blue grey dots | | | | |
| None | 73 (11) | 87 (13) | 87 (13) | 93 (14) |
| Mild | 20 (3) | 13 (2) | 13 (2) | 7 (1) |
| Moderate | 0 (0) | 0 (0) | 0 (0) | 0 (0) |
| Severe | 7 (1) | 0 (0) | 0 (0) | 0 (0) |
| Comedone-like outlets | | | | |
| None | 60 (9) | 60 (9) | 60 (9) | 73 (11) |
| Mild | 27 (4) | 33 (5) | 33 (5) | 27 (4) |
| Moderate | 13 (2) | 7 (1) | 7 (1) | 0 (0) |
| Severe | 0 (0) | 0 (0) | 0 (0) | 0 (0) |
| Horny pearls | | | | |
| None | 20 (3) | 20 (3) | 47 (7) | 33 (5) |
| Mild | 33 (5) | 60 (9) | 33 (5) | 47 (7) |
| Moderate | 13 (2) | 7 (1) | 7 (1) | 20 (3) |
| Severe | 33 (5) | 13 (2) | 13 (2) | 0 (0) |
| Scales | | | | |
| None | 47 (7) | 93 (14) | 80 (12) | 100 (15) |
| Mild | 40 (6) | 7 (1) | 20 (3) | 0 (0) |
| Moderate | 13 (2) | 0 (0) | 0 (0) | 0 (0) |
| Severe | 0 (0) | 0 (0) | 0 (0) | 0 (0) |
| Ice silvers structures | | | | |
| None | 33 (5) | 53 (8) | 53 (8) | 60 (9) |
| Mild | 27 (4) | 27 (4) | 40 (6) | 40 (6) |
| Moderate | 33 (5) | 20 (3) | 7 (1) | 0 (0) |
| Severe | 7 (1) | 0 (0) | 0 (0) | 0 (0) |

# Supplementary Table 2: Additional comparisons of dermatoscopic features at different time points.

|  | | Symptom at T1 (n=15) | | Fisher’s exact test | Symptom at T2 (n=15) | | Fisher’s exact test | Symptom at T3 (n=15) | | Binomial test |
| --- | --- | --- | --- | --- | --- | --- | --- | --- | --- | --- |
|  |  | N (%) | N (%) | p-value | N (%) | N (%) | p-value | N (%) | N (%) | p-value |
|  |  | No | Yes |  | No | Yes |  | No | Yes |  |
| Vascularisation at baseline | No | 0 (0) | 0 (0) | - | 0 (0) | 0 (0) | - | 0 (0) | 0 (0) | - |
|  | Yes | 1 (7) | 14 (83) | NS | 4 (27) | 11 (73) | NS | 7 (47) | 8 (53) | NS |
|  |  | No | Yes |  | No | Yes |  |  |  |  |
| Whitish plaques without structure at baseline | No | 0 (0) | 0 (0) | - | 0 (0) | 0 (0) | - | 0 (0) | 0 (0) | - |
|  | Yes | 2 (13) | 13 (87) | NS | 1 (7) | 14 (93) | NS | 1 (7) | 14 (93) | NS |
|  |  | No | Yes |  | No | Yes |  | No | Yes |  |
| Blue grey dots at baseline | No | 11 (73) | 0 (0) | - | 11 (73) | 0 (0) | - | 10 (67) | 1 (7) | - |
|  | Yes | 2 (13) | 2 (13) | NS | 2 (13) | 2 (13) | NS | 4 (27) | 0 (0) | NS |
|  |  | No | Yes |  | No | Yes |  | No | Yes |  |
| Purpuric lesions at baseline | No | 4 (27) | 0 (0) | - | 4 (27) | 0 (0) | - | 4 (27) | 0 (0) | - |
|  | Yes | 7 (47) | 4 (27) | NS | 6 (40) | 5 (33) | NS | 8 (53) | 3 (20) | NS |
|  |  | No | Yes |  | No | Yes |  | No | Yes |  |
| Comedone-like outlets at baseline | No | 9 (60) | 0 (0) | - | 9 (60) | 0 (0) | - | 9 (60) | 0 (0) | - |
|  | Yes | 0 (0) | 6 (40) | NS | 0 (0) | 6 (40) | NS | 2 (13) | 4 (27) | NS |
|  |  | No | Yes |  | No | Yes |  | No | Yes |  |
| Horny pearls at baseline | No | 2 (13) | 1 (7) | - | 3 (20) | 0 (0) | - | 2 (13) | 1 (7) | - |
|  | Yes | 0 (0) | 12 (80) | NS | 4 (27) | 8 (53) | NS | 3 (20) | 9 (60) | NS |
|  |  | No | Yes |  | No | Yes |  | No | Yes |  |
| Scales at baseline | No | 7 (47) | 0 (0) | - | 7 (47) | 0 (0) | - | 7 (47) | 0 (0) | - |
|  | Yes | 7 (47) | 1 (7) | p<0.05 | 5 (33) | 3 (20) | NS | 8 (53) | 0 (0) | p<0.05 |
|  |  | No | Yes |  | No | Yes |  | No | Yes |  |
| Ice silvers structures at baseline | No | 5 (33) | 0 (0) | - | 4 (27) | 1 (7) | - | 4 (27) | 1 (7) | - |
|  | Yes | 3 (20) | 7 (27) | NS | 4 (27) | 6 (40) | NS | 5 (33) | 5 (33) | NS |
|  |  | No | Yes |  | No | Yes |  | No | Yes |  |
| Whitish background at baseline | No | 0 (0) | 0 (0) | - | 0 (0) | 0 (0) | - | 0 (0) | 0 (0) | - |
|  | Yes | 0 (0) | 15 (100) | NS | 1 (7) | 14 (93) | NS | 3 (20) | 12 (80) | NS |

Analysis conducted using a binomial test with one-tailed t-test. Percentages were rounded to the nearest whole number.
Abbreviations: T1, 1-month post-treatment; T2, 3-months post-treatment; NA, not applicable; NS, not significant.

# Supplementary Table 3: Additional comparisons of sclerosis, leukoderma, hyperkeratosis, or purpuric lesions and abrasions or erosions at different time points.

|  | | Symptom at T1 (n=15) | | Fisher’s exact test | Symptom at T2 (n=15) | | Fisher’s exact test | Symptom at T3 (n=15) | | Binomial test |
| --- | --- | --- | --- | --- | --- | --- | --- | --- | --- | --- |
|  |  | N (%) | N (%) | p-value | N (%) | N (%) | p-value | N (%) | N (%) | p-value |
|  |  | No | Yes |  | No | Yes |  | No | Yes |  |
| Sclerosis at baseline | No | 0 (0) | 0 (0) | - | 0 (0) | 0 (0) | - | 0 (0) | 0 (0) | - |
|  | Yes | 0 (0) | 15 (100) | NS | 1 (7) | 14 (93) | NS | 2 (13) | 13 (87) | NS |
|  |  | No | Yes |  | No | Yes |  | No | Yes |  |
| Leukoderma (pallor) at baseline | No | 0 (0) | 0 (0) | - | 0 (0) | 0 (0) | - | 0 (0) | 0 (0) | - |
|  | Yes | 2 (13) | 13 (87) | NS | 2 (13) | 13 (87) | NS | 5 (33) | 10 (67) | NS |
|  |  | No | Yes |  | No | Yes |  | No | Yes |  |
| Hyperkeratosis at baseline | No | 8 (53) | 0 (0) | - | 8 (53) | 0 (0) | - | 8 (53) | 0 (0) | - |
|  | Yes | 6 (40) | 1 (7) | NS | 7 (47) | 0 (0) | p<0.05 | 7 (47) | 0 (0) | p<0.05 |
|  |  | No | Yes |  | No | Yes |  | No | Yes |  |
| Purpuric lesions, abrasions or erosions at baseline | No | 2 (13) | 0 (0) | - | 2 (13) | 0 (0) | - | 2 (13) | 0 (0) | - |
|  | Yes | 8 (53) | 5 (33) | NS | 9 (60) | 4 (27) | NS | 10 (67) | 3 (20) | p<0.05 |

Analysis conducted using a binomial test with one-tailed t-test. Percentages were rounded to the nearest whole number.
Abbreviations: T1, 1-month post-treatment; T2, 3-months post-treatment; NA, not applicable; NS, not significant.
